# Early autoantibody screening for type 1 diabetes: a Kuwaiti perspective on the advantages of multiplexing chemiluminescent assays

**DOI:** 10.3389/fimmu.2023.1273476

**Published:** 2023-11-29

**Authors:** Fahd Al-Mulla, Doha Alhomaidah, Mohamed Abu-Farha, Amal Hasan, Irina Al-Khairi, Rasheeba Nizam, Rawan Alqabandi, Hessa Alkandari, Jehad Abubaker

**Affiliations:** ^1^ Department of Translational Research, Dasman Diabetes Institute, Dasman, Kuwait; ^2^ Department of Population Health, Dasman Diabetes Institute, Dasman, Kuwait; ^3^ Department of Biochemistry and Molecular Biology, Dasman Diabetes Institute, Dasman, Kuwait; ^4^ Department of Genetic and Bioinformatics, Dasman Diabetes Institute, Dasman, Kuwait; ^5^ Special Services Department, Dasman Diabetes Institute, Dasman, Kuwait

**Keywords:** type 1 diabetes, autoantibody, population screening, multiplexing autoantibody screening, radio immune assay

## Abstract

Type 1 diabetes (T1D) incidence has increased globally over the last decades, alongside other autoimmune diseases. Early screening of individuals at risk of developing T1D is vital to facilitate appropriate interventions and improve patient outcomes. This is particularly important to avoid life-threatening diabetic ketoacidosis and hospitalization associated with T1D diagnosis. Additionally, considering that new therapies have been developed for T1D, screening the population and individuals at high risk would be of great benefit. However, adopting such screening approaches may not be feasible due to limitations, such as cost, adaptation of such programs, and sample processing. In this perspective, we explore and highlight the use of multiplexing chemiluminescent assays for T1D screening and emphasize on their advantages in detecting multiple autoantibodies simultaneously, maximizing efficiency, and minimizing sample volume requirements. These assays could be extremely valuable for pediatric populations and large-scale screening initiatives, providing a cost-efficient solution with increased diagnostic accuracy and deeper insights into T1D pathogenesis. Eventually, the adoption of such screening methods can help transform T1D diagnosis, especially in countries with high T1D prevalence, such as Kuwait, which will contribute to the development of novel therapeutic interventions, positively impacting the lives of those affected by T1D and other autoimmune diseases.

## 1 Type 1 diabetes prevalence

In recent decades, the incidence and prevalence of type 1 diabetes (T1D) have increased tremendously around the globe (1, 2). T1D is a multifactorial autoimmune disease characterized by immune-mediated destruction of pancreatic β-cells, resulting in a decline and cessation of insulin production. Although T1D has been associated with multiple genetic and non-genetic factors, its exact etiology remains poorly understood (3). More than 60 genetic susceptibility loci have been linked to T1D and nearly 50% of the inherent risk has been mapped to polymorphisms or specific haplotypes within the major histocompatibility complex (4, 5). In addition to this, several lifestyle factors, such as low vitamin D, exposure to toxins, dietary choices, and microbial dysbiosis are associated with the disease (6).

T1D is the most prevalent form of diabetes in children and young adults (1). Early diagnosis and treatment of T1D are critical for reducing its risk and severity. A young age of onset and extensive exposure to hyperglycemia may lead to serious secondary complications, affecting the kidneys, nerves, heart, or retina (7). Individuals with T1D are also at a higher risk of developing other autoimmune conditions, such as thyroiditis and celiac diseases (8). Moreover, one-third of children with T1D may experience diabetic ketoacidosis, accounting for an increased risk of morbidity and mortality (9).

## 2 Type 1 diabetes diagnosis

The presence of autoantibodies in peripheral blood, low C-peptide (< 0.3 nmol/l) levels, and hyperglycemia are the hallmark of Type 1 Diabetes. Around half of adult new onset T1D cases are primarily misdiagnosed as type 2 diabetes (10), indicating the significance of precise autoantibody testing for differentiating T1D from other forms of diabetes. Approximately 90% of the diagnosed T1D cases have one or more autoantibodies directed against pancreatic β-cells, including insulin autoantibodies (IAA), glutamic acid decarboxylase autoantibodies (GADA), islet antigen-2 autoantibodies (IA-2A), and zinc transporter 8 autoantibodies (ZnT8A) (11). IAA and GADA are the earliest autoantibodies present in circulation, appearing as early as two years of age in genetically high-risk children (12, 13). Seroconversion to a concurrent appearance of two or more autoantibodies in the circulation indicates a greater risk of developing T1D by the age of 18 years (13).

In summary, T1D development can be divided into various phases in genetically susceptible individuals. Several genetic risk loci associated with T1D have been identified in the human leukocyte antigen (HLA) complex, which plays an important role in the immune system. It is believed that T1D development cannot only be attributed to these risk loci but rather induced by an environmental trigger, such as viral infections or dietary components. As a result, the immune system triggers an attack on the body’s pancreatic β-cells. This attack is associated with the appearance of specific autoantibodies that are detectable in the blood before the onset of clinical symptoms, which can be a strong indicator of future T1D onset. Conventionally, this is termed “stage 1,” where two or more islet autoantibodies are present under normoglycemic conditions (**Table 1**). Stage 2 is marked by further destruction of pancreatic β-cells, leading to a progressive decline in insulin production and hyperglycemia.

**Table 1:** Stages of type 1 diabetes.

Stage	Description
Stage 1	The presence of two or more islet autoantibodies under normoglycemia (normal blood sugar levels). No clinical symptoms.
Stage 2	The presence of two or more islet autoantibodies under dysglycemia (impaired glucose tolerance). No clinical symptoms.
Stage 3	Clinical onset of type 1 diabetes, characterized by classic symptoms and hyperglycemia (high blood sugar levels).
Stage 4	Established type 1 diabetes, requiring lifelong insulin therapy and management to control blood sugar levels.

The onset of clinical symptoms occurs when approximately 80–90% of the β-cells have been destroyed and the body can no longer produce enough insulin to maintain normal blood sugar levels. This leads to clinical symptoms, such as excessive thirst, frequent urination, weight loss, and fatigue. Regarding diagnosis, T1D is typically diagnosed through blood tests that measure blood sugar levels and the presence of specific autoantibodies. Once diagnosed, the primary treatment for T1D is insulin therapy, which helps regulate blood sugar levels and prevent complications. Since circulating autoantibodies can be detected before the onset of clinical symptoms, they serve as early indicators for risk prediction and prognosis. The stages, as shown in **Table 1**, provide a framework for understanding T1D progression from the early presence of autoantibodies to the onset of clinical symptoms and the need for insulin therapy. Early identification of individuals at risk for T1D can help facilitate interventions to delay or prevent the onset of the disease.

One of the benchmark techniques for the detection and quantification of autoantibodies is radio-binding assay; however, its use in clinical practice is limited due to radioactive isotope requirements (14). Quantitative enzyme-linked immunosorbent assays (ELISAs) are laborious, with numerous wash protocols and limited scalability. Recently, there has been a growing interest in chemiluminescence methods to detect the presence of autoantibodies, which offers the unique advantages of reliable and rapid detection of multiple autoantibodies at a low cost.

## 3 Type 1 diabetes prevention and treatment

In T1D, the body’s immune system, specifically T-cells, attack and destroy insulin-producing pancreatic β-cells. Teplizumab, also known as Tzield, is a humanized monoclonal antibody that targets the CD3 receptor on T-cells. It is being investigated as a potential therapy for the prevention and treatment of T1D. Teplizumab works by binding to the CD3 receptor, which modulates T-cell activation and, in so doing, helps preserve the remaining β-cell function in individuals with T1D or those at high risk of developing the disease based on certain criteria.

In a key clinical trial published in the *New England Journal of Medicine* (Herold et al., 2019), teplizumab was found to delay the onset of T1D in high-risk individuals with a family history of the disease and at least two detectable autoantibodies (15). The median time to T1D diagnosis was significantly longer in the teplizumab group (48.4 months) as compared to the placebo group (24.4 months) (15). Moreover, the risk of developing T1D was reduced by 59% in the teplizumab group, as compared to the placebo group. These results suggest that teplizumab could potentially be a useful therapeutic option for the prevention of T1D, and early intervention in indiviudals with high-risk of T1D. Teplizumab was finally approved on November 17th, 2022 for adults and children aged 8 years and older with stage 2 type 1 diabetes (16).

Another treatment that has shown promise is the repurposed use of verapamil for the treatment of T1D. Verapamil is a calcium channel blocker that is approved by the Food and Drug Administration (FDA) for treating cardiovascular conditions, such as hypertension, angina, and arrhythmias. Verapamil inhibits L-type calcium channels, thereby reducing calcium channel influx, which prevents β-cell impairment that arises due to chronic increases in calcium levels (17). Currently, the drug is known to act by inhibiting the transcription of thioredoxin-interacting protein (TXNIP), whose accumulation is known to increase oxidative toxicity in the pancreas (18). TXNIP downregulation leads to a decrease in β-cell apoptosis, subsequently improving β-cell function and ameliorating insulin production and secretion (17–19). Studies conducted on both mice models and patients with T1D have found that verapamil improved β-cell function and survival, ameliorated glucose sensitivity and homeostasis, and increased insulin levels (17, 20) Moreover, in clinical trials, it was observed that verapamil reduced the risk of new-onset diabetes when compared to other calcium channel blockers (21).

## 4 Autoantibody screening

Autoantibody screening is a crucial tool for predicting and diagnosing T1D, as the presence of autoantibodies indicates an autoimmune response against the insulin-producing pancreatic β-cells (22). There are several types of autoantibody screening methods used for T1D, including (22):

1. Radioimmunoassay (RIA): This is a highly sensitive and specific method that uses radioactive-labeled antigens to detect autoantibodies. RIA has been the gold standard for autoantibody detection of T1D for several years (23). However, it involves the use of radioactive materials, making it less suitable for routine clinical use.

2. ELISA: This widely used method employs enzyme-labeled antigens or antibodies to detect autoantibodies. Although it is a cost-effective and robust method suitable for large-scale screening, it can be less sensitive and specific, as compared to RIA (24).

3. Electrochemiluminescence (ECL) assay: This technique uses chemiluminescent labels and electrical signals to measure autoantibodies, offering high sensitivity and specificity. ECL assays are gaining popularity due to their ability to detect low concentrations of autoantibodies and their potential for simultaneously detecting multiple autoantibodies (multiplexing) (25).

4. Multiplex immunoassays: These assays are designed to detect multiple autoantibodies simultaneously, improving efficiency and reducing sample volume requirements. Multiplex immunoassays can be performed using various platforms, such as bead-based assays, planar arrays, and chemiluminescent assays (26).

## 5 Screening strategies

Various autoantibody screening strategies can be employed to identify individuals at risk of developing T1D at various stages of life and within different populations (27). These strategies include (27):

1. Screening relatives of patients with T1D: As T1D has a genetic component, first-degree relatives (e.g., siblings, children, and parents) of individuals with T1D have an increased risk of developing the disease. Screening these relatives for islet autoantibodies can help identify those at risk and facilitate early intervention.

2. General population screening: This strategy involves screening a broader population for autoantibodies, regardless of family history. Although it is less targeted than screening relatives, this approach can identify at-risk individuals who would not be detected through familial screening alone. However, this may be less cost-effective due to the lower prevalence of T1D in the general population.

3. Birth cohort screening: This approach involves the prospective screening of newborns or young children for islet autoantibodies. Early identification of autoantibody-positive children can help monitor their disease progression and implement preventive measures before T1D onset. Birth cohort studies also provide valuable insights into the natural history of the disease and potential environmental triggers.

4. High-risk group screening: This strategy targets the group of individuals known to have a higher risk of developing T1D, such as those with specific genetic markers (e.g., HLA genotypes), other autoimmune diseases, or certain demographic characteristics (e.g., specific age groups or ethnicities). This targeted approach can increase the likelihood of identifying individuals at risk and is potentially more cost-effective than general population screening.

Each screening strategy has its advantages and limitations, and the choice of approach depends on factors, such as cost, resources, and the specific goals of the screening program. Ultimately, the early identification of at-risk individuals through autoantibody screening can enable timely interventions to delay or prevent T1D onset (27).

## 6 Advantages of early screening strategies

Early screening for T1D offers several advantages for at-risk individuals and the healthcare system (27, 28). These include:

1. Early intervention: Identifying individuals with islet autoantibodies before the clinical onset of T1D allows for the timely implementation of preventive measures and treatments, such as lifestyle modifications, immunotherapies, or other experimental interventions, potentially delaying or preventing disease onset.

2. Better glycemic control: Early detection of T1D enables prompt initiation of insulin therapy and other diabetes management strategies, leading to improved glycemic control. Better glycemic control translates to a reduced risk of long-term complications, such as retinopathy, nephropathy, neuropathy, and cardiovascular disease.

3. Preserved residual β-cell function: Initiating early treatment may help preserve residual β-cell function, which is associated with better glycemic control, fewer hypoglycemic events, and a reduced need for exogenous insulin.

4. Psychological benefits: Early identification of those at risk of T1D can help individuals and their families prepare for potential lifestyle changes, adjust to the idea of living with diabetes, and seek appropriate psychological support if needed.

5. Enhanced understanding of T1D pathogenesis: Large-scale screening programs and longitudinal studies can provide valuable insights into the natural history of the disease, the role of environmental factors, and the interaction between genetic and environmental risk factors.

6. Cost-effectiveness: Early intervention and improved disease management can ease the burden on healthcare by reducing costs associated with diabetes complications and hospitalizations. Having said that, the cost-effectiveness of screening depends on the specific screening strategy and target population.

It is essential to weigh these potential benefits of T1D screening against the possible psychological, social, and financial impacts on individuals and their families. Informed decision-making and appropriate support systems are crucial for the effective implementation of early screening programs (14, 29, 30).

## 7 Multiplexing autoantibody screening

Multiplexing autoantibody screening for T1D offers several advantages over traditional single autoantibody assays, making it an attractive option for large-scale screening programs and research applications (26). The advantages include:

1. Increased efficiency: Multiplexing allows for the simultaneous detection of multiple autoantibodies in a single assay, streamlining the screening process and reducing the time and labor required to analyze multiple samples.

2. Reduced sample volume: Multiplex assays often require smaller sample volumes than single autoantibody tests, making them more suitable for pediatric populations or individuals with limited blood sample availability.

3. Cost-effectiveness: By measuring multiple autoantibodies in a single assay, multiplexing can reduce the cost per sample, making it a more cost-effective option for large-scale screening initiatives.

4. Enhanced diagnostic accuracy: Detecting multiple autoantibodies can increase the sensitivity and specificity of T1D risk assessment, enabling better identification of at-risk individuals and minimizing false-positive or false-negative results.

5. Comprehensive risk assessment: Some individuals with T1D may have only one detectable autoantibody, while others may have multiple autoantibodies. Multiplexing allows for a more comprehensive assessment of autoantibody profiles, enabling a better understanding of an individual’s risk for disease progression.

6. Improved research capabilities: Multiplex autoantibody assays can provide more extensive data on the autoantibody profile of individuals with T1D or at risk for the disease, which can be valuable for researchers studying the pathogenesis of T1D, the role of autoantibodies in disease progression, and potential therapeutic interventions.

Despite these advantages, it is essential to ensure that multiplex autoantibody assays are carefully validated and optimized for sensitivity, specificity, and reproducibility before being adopted for large-scale screening or research applications.

## 8 Kuwait multiplexing chemiluminescent autoantibody screening:

Our team has been working diligently to develop a multiplexing chemiluminescent autoantibody screening kit for the simultaneous detection of three key autoantibodies associated with T1D, including GADA, IA-2A, and ZnT8A. This innovative screening approach offers several benefits over traditional single autoantibody assays as mentioned above including increased efficiency, reduced sample volume requirements, cost-effectiveness, enhanced diagnostic accuracy, and comprehensive risk assessment. Throughout the development process, we focused on optimizing the assay’s sensitivity, specificity, and reproducibility. Our goal is to create a reliable and accurate tool for identifying individuals at risk of T1D and facilitating early intervention strategies to prevent or delay disease onset. The study was conducted in accordance with the Declaration of Helsinki and approved by the Institutional Review Board at Dasman Diabetes Institute (protocol code RA-MOH-2021-016 and date of approval: Sep 11-2021).

**Figure 1:**
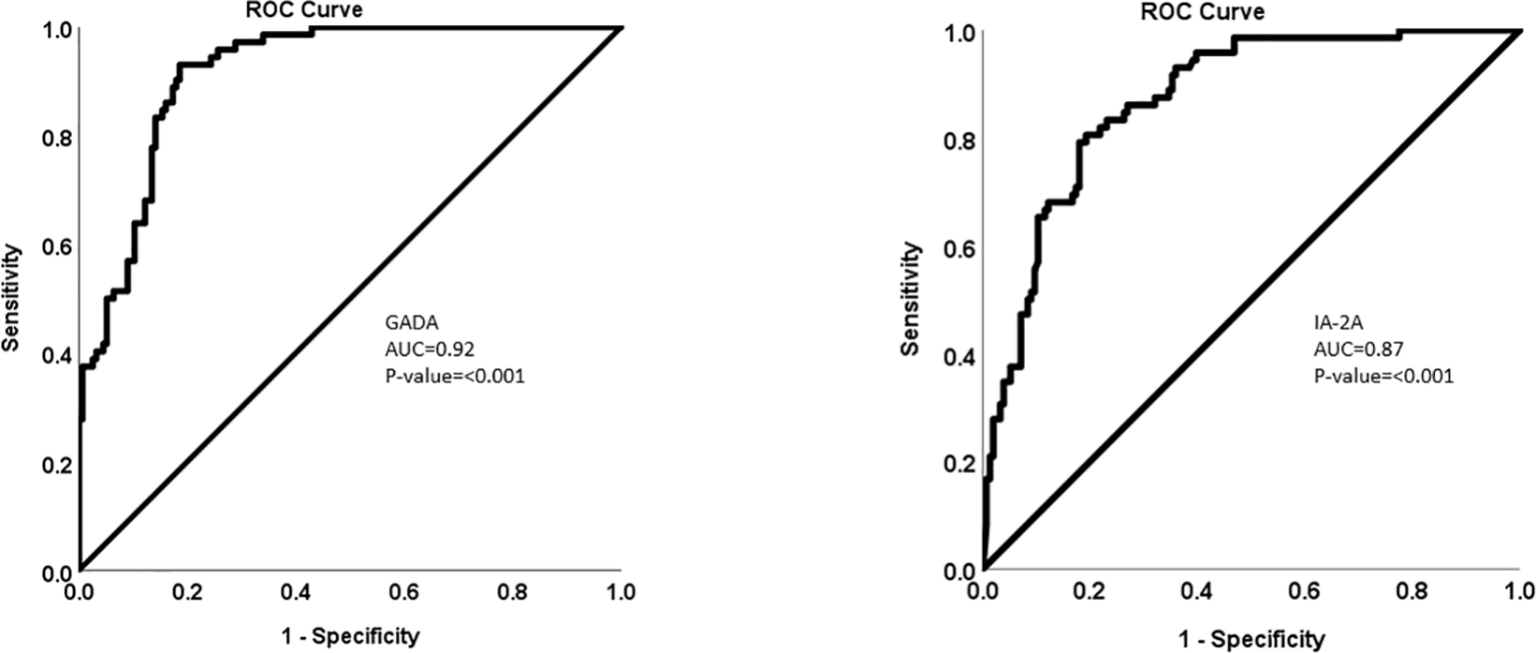
ROC analysis for the multiplexing data for both IA2A and GADA.

**Figure 2:**
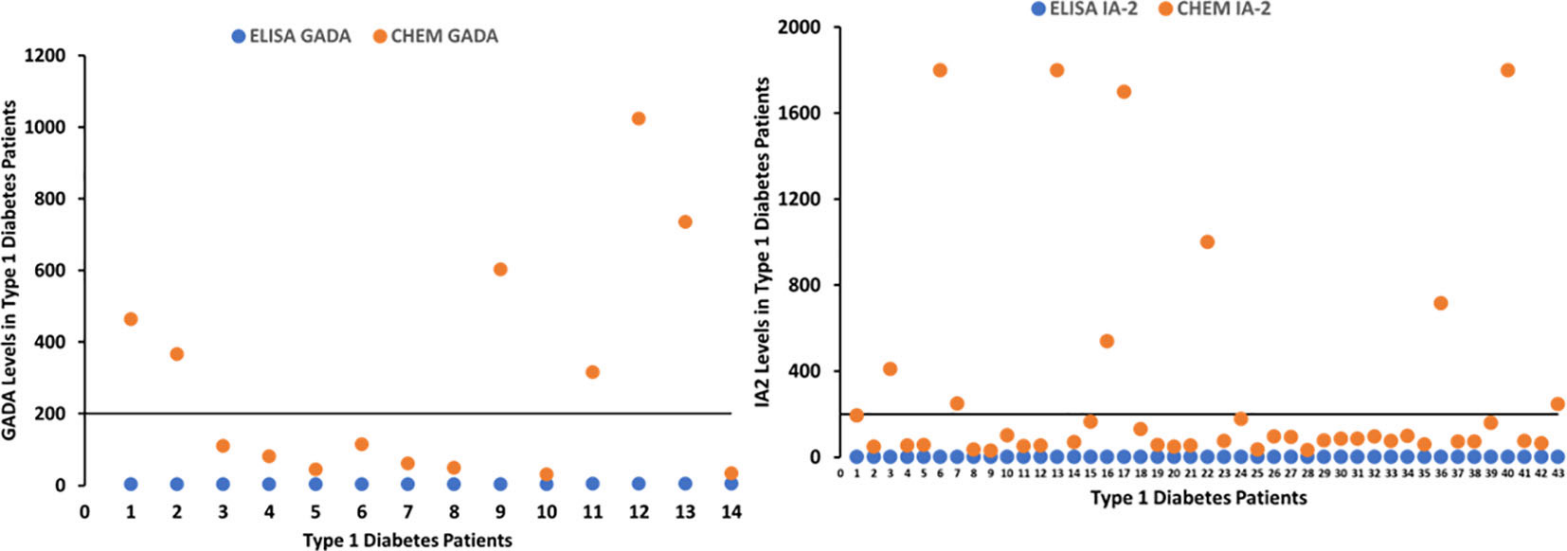
Graphs depicting patients with T1D positive for GADA and IA-2A detected by chemiluminescent assay, whose values were negative with ELISA.

The multiplexing chemiluminescent autoantibody screening kit that we are developing has the potential to transform the way T1D screening is performed, offering a more efficient and cost-effective solution for large-scale screening programs and research applications. By enabling the simultaneous detection of GADA, IA-2A, and ZnT8A, our screening kit can provide a more comprehensive assessment of an individual’s risk for T1D and contribute to an improved understanding of the disease’s pathogenesis, paving the way for the development of novel therapeutic interventions. Refer to **Figures 1** and **2**.

The development of the Q-Plex™ Human Diabetes Panel 1 (2-Plex) was completed by Quansys Biosciences (Logan, UT, USA) with ZnT8A under development. Multiplexed plates were created by printing the proteins onto a high-binding polystyrene clear-bottom plate, that was subsequently blocked to prevent nonspecific binding. Breifly, the manufacturer’s protocol for running the assay were as follows:

50 µL of plasma (at a 1:200 dilution in sample diluent), calibrator, or controls were pipetted into the wells and left to incubate for 45 minutes. The plate was then washed three times to remove any unbound IgG, and 50 µL of a biotinylated anti-human IgG mixture was added to each well for 30 minutes. After an additional 3 washes to remove unbound biotinylated antibody, 50 µL of streptavidin-horseradish peroxidase (SHRP) was added to each well for 15 minutes. The plates were washed an additional six times. The remaining amount of SHRP on each location of the array is proportional to the initial amount of human IgG antibody reactive to GAD-65, IA-2, and total human IgG (positive control) that was captured. To measure the amount of conjugated enzyme on each location of the array, a chemiluminescent substrate was added, which reacts with the SHRP. The resulting chemiluminescent signal was then measured, which is proportional to the amount of human IgG antibodies reactive to GAD-65 and IA-2 in the original sample. By comparing the signal to the wells loaded with a calibrator, the quantity of bound IgG per sample was calculated.

Using the above protocol several metrics were used to validate the assay.

The upper limit of quantification (ULOQ) on initial lots was determined to be 4650 U/mL for GAD-65 and 1960 U/mL for IA-2. The lower limit of quantification (LLOQ) was 0.099 U/mL for GAD-65 and 0.012 U/mL for IA-2. Intra-array cross reactivity was tested by separately spiking IA-2 into the sample diluent at 10µg/mL and GAD65 at 1µg/mL. Corresponding signal depression (>50%) on 3 unique samples was seen for both analytes. Intra-assay precision was measured using 20 replicates of 3 samples on 3 kits and found to have an average percent coefficient of variation (%CV) of 13% for GAD 65 and 15% for IA-2. Inter-assay precision was measured using at least 4 replicates of 3 samples on 8 kits and found to have an average %CV of 12% for GAD65 and 17% for IA-2. Sample correlation to predicate assays run at the Dasman Diabetes Institute (DDI) on 20 samples showed 0.80 for GAD-65 and 0.95 for IA-2. Analytes in samples diluted between 1 to 200 and 1 to 3200 exhibited a percent linearity between 80-120%. Bilirubin, lipids, and human antimouse antibodies do not interfere with the assay at concentrations expected in clinical samples. Hemoglobin significantly interacts with both analytes at a concentration above 1.25 mg/mL. Hemolyzed samples should not be used in the array. All wells used in a single kit should be loaded within 5 minutes upon beginning the assay. Shelf-life stability testing is ongoing.

The T1D screening kit shows great promise especially with the development of new medications but comes with several limitations. Demographic factors like age and gender influence the prevalence of T1D autoantibodies, necessitating careful result interpretation across diverse populations. The risk of false positives and negatives, exacerbated by factors like hemolysis, requires strict quality control. The kit’s cost-effectiveness should be evaluated, accounting for potential savings from early intervention. However, such a program can be potentially effective at reducing the burden of T1D.

## 9 Conclusions

In conclusion, early screening of individuals at risk for developing T1D is crucial for timely intervention and potentially preventing or delaying the onset of the disease. Identifying at-risk individuals enables healthcare providers to implement preventive measures, tailor treatment plans, and improve overall patient outcomes. The development and implementation of efficient and reliable screening methods are essential for achieving this goal.

Multiplexing chemiluminescent assays offer significant advantages for T1D screening, particularly in cases where low blood volume samples are required. By enabling the simultaneous detection of multiple autoantibodies, these assays provide a comprehensive assessment of an individual’s risk for T1D while maximizing efficiency. This makes multiplexing chemiluminescent assays particularly well-suited for pediatric populations and large-scale screening initiatives.

Moreover, adopting multiplexing chemiluminescent assays for T1D screening can lead to cost savings, enhanced diagnostic accuracy, and a more extensive understanding of the disease’s pathogenesis. Ultimately, widespread implementation of such advanced screening methods has the potential to revolutionize T1D risk assessment and contribute to the development of novel therapeutic interventions, improving the lives of numerous individuals affected by or at risk from this chronic condition.

## Data availability statement

The original contributions presented in the study are included in the article/**Supplementary Material**. Further inquiries can be directed to the corresponding author.

## Ethics statement

The studies involving humans were approved by Dasman Diabetes Institute Ethical Review Comittee. The studies were conducted in accordance with the local legislation and institutional requirements. Written informed consent for participation in this study was provided by the participants’ legal guardians/next of kin.

## Author contributions

FA: Conceptualization, Supervision, Writing – original draft, Writing – review & editing. DA: Methodology, Supervision, Writing – review & editing. MA: Conceptualization, Data curation, Methodology, Writing – original draft. AH: Writing – review & editing. IA: Data curation, Formal Analysis, Methodology, Writing – original draft. RN: Investigation, Methodology, Writing – original draft. RA: Project administration, Writing – review & editing. HA: Funding acquisition, Methodology, Project administration, Writing – review & editing. JA: Formal Analysis, Methodology, Supervision, Writing – review & editing.

## References

[1] Mobasseri M Shirmohammadi M Amiri T Vahed N Hosseini Fard H Ghojazadeh M. Prevalence and incidence of type 1 diabetes in the world: a systematic review and meta-analysis. Health promotion Perspect (2020) 10:98–115. doi: 10.34172/hpp.2020.18 PMC7146037 32296622

[2] Ogurtsova K da Rocha Fernandes JD Huang Y Linnenkamp U Guariguata L Cho NH. IDF Diabetes Atlas: Global estimates for the prevalence of diabetes for 2015 and 2040. Diabetes Res Clin Pract (2017) 128:40–50. doi: 10.1016/j.diabres.2017.03.024 28437734

[3] DiMeglio LA Evans-Molina C Oram RA. Type 1 diabetes. Lancet (2018) 391:2449–62. doi: 10.1016/S0140-6736(18)31320-5 PMC6661119 29916386

[4] Bakay M Pandey R Grant SFA Hakonarson H. The genetic contribution to type 1 diabetes. Curr Diabetes Rep (2019) 19:116. doi: 10.1007/s11892-019-1235-1 31686270

[5] Nguyen C Varney MD Harrison LC Morahan G. Definition of high-risk type 1 diabetes HLA-DR and HLA-DQ types using only three single nucleotide polymorphisms. Diabetes (2013) 62:2135–40. doi: 10.2337/db12-1398 PMC3661605 23378606

[6] Quinn LM Wong FS Narendran P. Environmental determinants of type 1 diabetes: from association to proving causality. Front Immunol (2021) 12:737964. doi: 10.3389/fimmu.2021.737964 34659229 PMC8518604

[7] Tommerdahl KL Shapiro ALB Nehus EJ Bjornstad P. Early microvascular complications in type 1 and type 2 diabetes: recent developments and updates. Pediatr Nephrol (2022) 37:79–93. doi: 10.1007/s00467-021-05050-7 33852054 PMC8527882

[8] Holt RIG DeVries JH Hess-Fischl A Hirsch IB Kirkman MS Klupa T. Correction to: The management of type 1 diabetes in adults. A consensus report by the American Diabetes Association (ADA) and the European Association for the Study of Diabetes (EASD). Diabetologia (2022) 65:255. doi: 10.1007/s00125-021-05600-6 34851428 PMC8895052

[9] Dabelea D Rewers A Stafford JM Standiford DA Lawrence JM Saydah S. Trends in the prevalence of ketoacidosis at diabetes diagnosis: the SEARCH for diabetes in youth study. Pediatrics (2014) 133:e938–945. doi: 10.1542/peds.2013-2795 PMC4074618 24685959

[10] Hope SV Wienand-Barnett S Shepherd M King SM Fox C Khunti K. Practical Classification Guidelines for Diabetes in patients treated with insulin: a cross-sectional study of the accuracy of diabetes diagnosis. Br J Gen Pract J R Coll Gen Practitioners (2016) 66:e315–322. doi: 10.3399/bjgp16X684961 PMC4838443 27080317

[11] McLaughlin KA Richardson CC Ravishankar A Brigatti C Liberati D Lampasona V. Identification of tetraspanin-7 as a target of autoantibodies in type 1 diabetes. Diabetes (2016) 65:1690–8. doi: 10.2337/db15-1058 26953162

[12] Krischer JP Lynch KF Schatz DA Ilonen J Lernmark Å. Hagopian WA. The 6 year incidence of diabetes-associated autoantibodies in genetically at-risk children: the TEDDY study. Diabetologia (2015) 58:980–7. doi: 10.1007/s00125-015-3514-y PMC4393776 25660258

[13] Ziegler AG Rewers M Simell O Simell T Lempainen J Steck A. Seroconversion to multiple islet autoantibodies and risk of progression to diabetes in children. Jama (2013) 309:2473–9. doi: 10.1001/jama.2013.6285 PMC4878912 23780460

[14] Yu L Zhao Z Steck AK. T1D Autoantibodies: room for improvement? Curr Opin Endocrinol Diabetes Obes (2017) 24:285–91. doi: 10.1097/MED.0000000000000348 PMC5815324 28509692

[15] Herold KC Bundy BN Long SA Bluestone JA DiMeglio LA Dufort MJ. An anti-CD3 antibody, teplizumab, in relatives at risk for type 1 diabetes. N Engl J Med (2019) 381:603–13. doi: 10.1056/NEJMoa1902226 PMC6776880 31180194

[16] Hirsch JS. FDA approves teplizumab: a milestone in type 1 diabetes. Lancet Diabetes Endocrinol (2023) 11:18. doi: 10.1016/S2213-8587(22)00351-5 36436528

[17] Ovalle F Grimes T Xu G Patel AJ Grayson TB Thielen LA. Verapamil and beta cell function in adults with recent-onset type 1 diabetes. Nat Med (2018) 24:1108–12. doi: 10.1038/s41591-018-0089-4 PMC6092963 29988125

[18] Xu G Chen J Jing G Shalev A. Preventing beta-cell loss and diabetes with calcium channel blockers. Diabetes (2012) 61:848–56. doi: 10.2337/db11-0955 PMC3314354 22442301

[19] Borowiec AM Wlaszczuk A Olakowska E Lewin-Kowalik J. TXNIP inhibition in the treatment of diabetes. Verapamil as a novel therapeutic modality in diabetic patients. Med Pharm Rep (2022) 95:243–50. doi: 10.15386/mpr-2187 PMC9387585 36060506

[20] Forlenza GP McVean J Beck RW Bauza C Bailey R Buckingham B. Effect of verapamil on pancreatic beta cell function in newly diagnosed pediatric type 1 diabetes: A randomized clinical trial. JAMA (2023) 329:990–9. doi: 10.1001/jama.2023.2064 PMC9960020 36826844

[21] Yin T Kuo SC Chang YY Chen YT Wang KK. Verapamil use is associated with reduction of newly diagnosed diabetes mellitus. J Clin Endocrinol Metab (2017) 102:2604–10. doi: 10.1210/jc.2016-3778 28368479

[22] So M Speake C Steck AK Lundgren M Colman PG Palmer JP. Advances in type 1 diabetes prediction using islet autoantibodies: beyond a simple count. Endocr Rev (2021) 42:584–604. doi: 10.1210/endrev/bnab013 33881515

[23] Bingley PJ Williams AJ. Islet autoantibody testing: an end to the trials and tribulations? Diabetes (2013) 62:4009–11. doi: 10.2337/db13-1445 PMC3837043 24264404

[24] Lampasona V Pittman DL Williams AJ Achenbach P Schlosser M Akolkar B. Islet autoantibody standardization program 2018 workshop: interlaboratory comparison of glutamic acid decarboxylase autoantibody assay performance. Clin Chem (2019) 65:1141–52. doi: 10.1373/clinchem.2019.304196 PMC8936135 31409598

[25] Miao D Steck AK Zhang L Guyer KM Jiang L Armstrong T. Electrochemiluminescence assays for insulin and glutamic acid decarboxylase autoantibodies improve prediction of type 1 diabetes risk. Diabetes Technol Ther (2015) 17:119–27. doi: 10.1089/dia.2014.0186 PMC4321773 25562486

[26] Zhao Z Miao D Michels A Steck A Dong F Rewers M. A multiplex assay combining insulin, GAD, IA-2 and transglutaminase autoantibodies to facilitate screening for pre-type 1 diabetes and celiac disease. J Immunol Methods (2016) 430:28–32. doi: 10.1016/j.jim.2016.01.011 26809048 PMC5851776

[27] Sims EK Besser REJ Dayan C Geno Rasmussen C Greenbaum C Griffin KJ. Screening for type 1 diabetes in the general population: A status report and perspective. Diabetes (2022) 71:610–23. doi: 10.2337/dbi20-0054 PMC9114719 35316839

[28] Zhang D Huang J Hu J. Improved diagnosis of type-1 diabetes mellitus using multiplexed autoantibodies ELISA array. Anal Biochem (2022) 649:114722. doi: 10.1016/j.ab.2022.114722 35537484

[29] Jia X Yu L. Effective assay technologies fit for large-scale population screening of type 1 diabetes. Front Clin Diabetes Healthc (2022) 3:1034698. doi: 10.3389/fcdhc.2022.1034698 36992730 PMC10012058

[30] He L Jia X Rasmussen CG Waugh K Miao D Dong F. High-throughput multiplex electrochemiluminescence assay applicable to general population screening for type 1 diabetes and celiac disease. Diabetes Technol Ther (2022) 24:502–9. doi: 10.1089/dia.2021.0517 PMC9464081 35238620

